# Mutual exclusive gene expression reveals a stress-induced compensatory role of taurine uptake in dilated cardiomyopathy

**DOI:** 10.1093/bib/bbaf631.063

**Published:** 2025-12-12

**Authors:** Jinpu Cai, Xiaorui Liu, Cheng Wang, Yibo Zhang, Luqi Yang, Bingyi Li, Luting Zhou, Ziqi Rong, Haoyang Liu, Linkang He, Xinzhu Jiang, Hui Cheng, Yu Zhao, Bing He, Jianhua Yao, Hongbin Shen, Shyam Prabhakar, Qiuyu Lian, Liang Chen, Hongyi Xin

**Affiliations:** Global College, SJTU, Shanghai, China; Fuwai Hospital, Beijing, China; Global Institute of Future Technology, SJTU, Shanghai, China; Fuwai Hospital, Beijing, China; Global Institute of Future Technology, SJTU, Shanghai, China; School of Biomedical Engineering, SJTU, Shanghai, China; Global College, SJTU, Shanghai, China; Bioinformatics Interdepartmental Program, UCLA, CA, United States; Global Institute of Future Technology, SJTU, Shanghai, China; Fuwai Hospital, Beijing, China; Global College, SJTU, Shanghai, China; Global College, SJTU, Shanghai, China; Tencent, Shenzhen, China; Tencent, Shenzhen, China; Tencent, Shenzhen, China; School of Automation and Intelligent Sensing, SJTU, Shanghai, China; A*STAR, Singapore; University of Cambridge, Cambridge, United Kingdom; Fuwai Hospital, Beijing, China; Global Institute of Future Technology, SJTU, Shanghai, China

## Abstract

Mutually exclusive gene expression, where gene pairs are expressed in strict alternation within individual cells, reflects fundamental inter-gene regulatory mechanisms and can reveal shifts in transcriptional programs during development or disease. Detecting such patterns is critical for resolving rare cellular subpopulations, temporally discrete states along pseudotime, and spatially segregated neighborhoods in single-cell and spatial multi-omics data. However, the sparsity and dropout inherent to single-cell data make mutually exclusive expression difficult to detect, leading conventional feature selection methods to overlook subtle yet functionally important genes.

We present MULE, an unbiased framework that systematically organizes collective mutual exclusivity into a hierarchical taxonomy. Applying MULE to cardiac datasets, we uncovered robust upregulation of SPOCK1 and SLC6A6 in dilated cardiomyopathy, previously obscured by the inability to resolve pathological cardiomyocytes. In vivo and in vitro experiments demonstrated that stress-induced SLC6A6 upregulation serves a cardiomyocyte self-protective mechanism. Taurine supplementation reduced oxidative stress, restored calcium homeostasis, prevented cell death, and improved cardiac function post-injury. These findings elucidate a novel cardiomyocyte stress response and highlight the therapeutic promise of taurine supplementation for the treatment of dilated cardiomyopathy.

